# Extended Multiple Aperture Mapdrift-Based Doppler Parameter Estimation and Compensation for Very-High-Squint Airborne SAR Imaging

**DOI:** 10.3390/s19010213

**Published:** 2019-01-08

**Authors:** Zhichao Zhou, Yinghe Li, Yan Wang, Linghao Li, Tao Zeng

**Affiliations:** 1 School of Information and Electronics, Beijing Institute of Technology, Beijing 100081, China; zcz1024@foxmail.com (Z.Z.); lilinghaodhd@foxmail.com (L.L.); zengtao@bit.edu.cn (T.Z.); 2 Beijing Key Laboratory of Embedded Real-Time Information Processing Technology, Beijing 100081, China; 3 Beijing Institute of Radio Measurement, Beijing 100081, China; liyinghe@bit.edu.cn

**Keywords:** Doppler parameter estimation and compensation (DPEC), extended multiple aperture mapdrift (EMAM), very-high-squint airborne SAR imaging, spatial variance, the derivative of the Doppler rate

## Abstract

Doppler parameter estimation and compensation (DPEC) is an important technique for airborne SAR imaging due to the unpredictable disturbance of real aircraft trajectory. Traditional DPEC methods can be only applied for broadside, small- or medium-squint geometries, as they at most consider the spatial variance of the second-order Doppler phase. To implement the DPEC in very-high-squint geometries, we propose an extended multiple aperture mapdrift (EMAM) method in this paper for better accuracy. This advantage is achieved by further estimating and compensating the spatial variation of the third-order Doppler phase, i.e., the derivative of the Doppler rate. The main procedures of the EMAM, including the steps of sub-view image generation, sliding-window-based cross-correlation, and image-offset-based Doppler parameter estimation, are derived in detail, followed by the analyses for the EMAM performance. The presented approach is evaluated by both computer simulations and real airborne data.

## 1. Introduction

**Figure 1:**
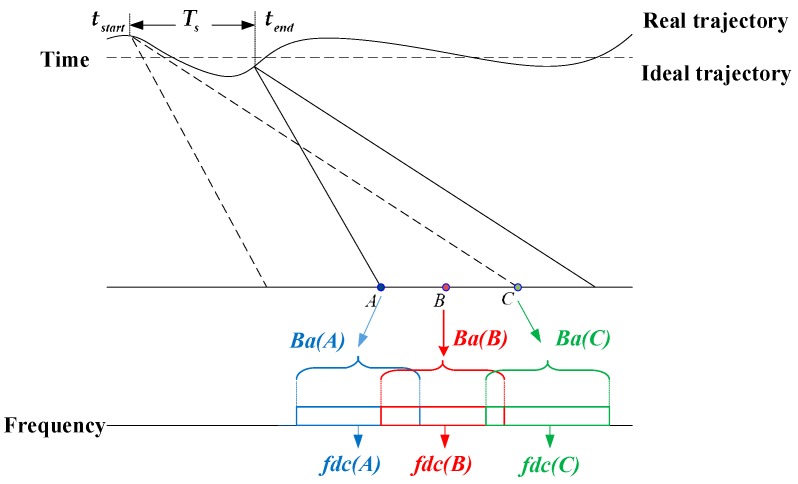
The illumination of the spatially-variant Doppler histories of targets at different positions for the very-high-squint (VHS) airborne SAR imaging. $T_{s}$ is the aircraft motion time. $t_{start}$ and $t_{end}$ are the time start and end, respectively. $B_{a}$ is the Doppler bandwidth. $f_{dc}$ is the Doppler centroid.

Airborne synthetic aperture radar (SAR) [1,2,3,4,5] is an all-weather and all-day microwave imaging sensor that can provide two-dimensional high-resolution images of illuminated regions. High-squint airborne SAR [6] is necessary for inverting target electromagnetic scattering characteristics in one track of observation and, therefore, is significant for accurate target identification [7,8,9,10]. The larger the squint angle, the more flexible the data acquisition and hence the more information a single observation can achieve. For the very-high-squint (VHS) airborne SAR imaging, targets at different positions have spatially-variant Doppler histories, as shown in Figure 1. While in range (along the direction of electromagnetic wave propagation), the spatial variance can be easily estimated and compensated by range blocking, it is not that convenient to estimate and compensate the azimuth spatially-variant Doppler parameters (along the direction perpendicular to the direction of electromagnetic wave propagation). Note that the spatial variance used below refers to the azimuth spatial variance if without additional denotations.

One of the main challenges of the airborne SAR imaging is the Doppler parameter estimation and compensation (DPEC) because the positioning, velocity, and angle information provided by the onboard inertial navigation system are generally not accurate enough for the high-squint high-resolution imaging [11]. Moreover, the real aircraft trajectories often deviate from the ideal trajectories due to unexpected disturbances [12,13,14,15] as shown in Figure 1, which leads to the Doppler parameter errors. If the spatially-variant Doppler parameter estimation (DPE) is not considered and left compensated, the SAR image quality will be seriously deteriorated. Thus, it is necessary to perform echo-based DPE to ensure good focusing performance [16,17,18,19,20,21]. For the VHS airborne SAR, the DPEC is more challenging because of its complex spatially-variant characteristics.

**Figure 2:**
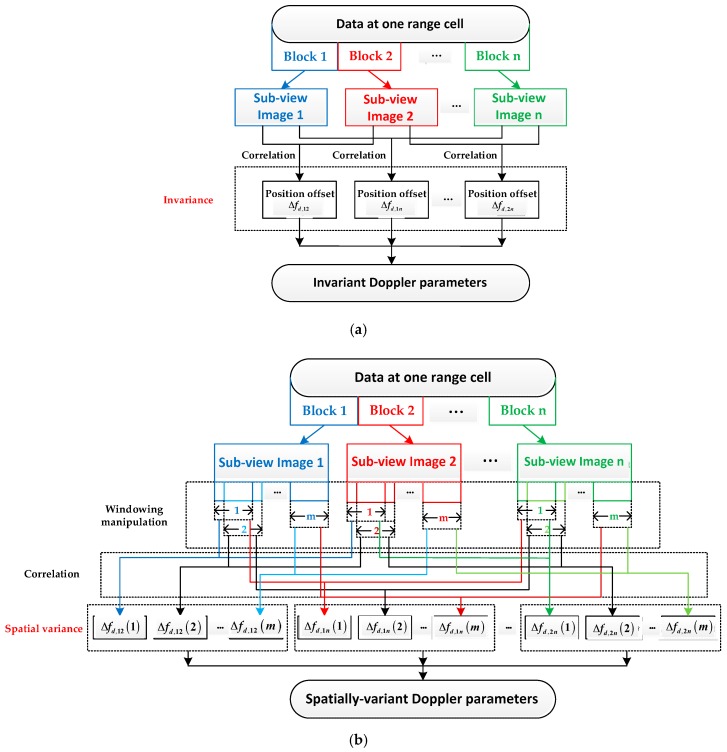
The illuminations of the basic MAM method and the EMAM method. (**a**) The basic MAM method; (**b**) the EMAM method. $\Delta f_{d,ij}$ is the position offset between the sub-i image and sub-j image.

Traditionally, the DPE can be implemented by the multiple aperture mapdrift (MAM) via the azimuth multi-view processing. The basic MAM method (as shown in Figure 2a) [22,23] assumes that the Doppler parameters do not change with respect to target positions. In this case, the estimated Doppler parameters are the averaged results of the real ones. Although such approximation is valid for the broadside or small-squint SAR imaging, it is no longer valid for the high-squint cases because the spatially-dependent components of the DPE will seriously degrade the image quality if left uncompensated. Although there exist some methods for the spatially-variant DPE, such as the improved MAM (IMAM) method [24,25], their accuracy is limited as they only deal with the spatial variance of the second-order Doppler phase. In the VHS case, for instance, with a 70-degree squint angle [26], the less accurate DPEC methods will lead to serious image quality degradation.

Aiming at implementing accurate enough DPEC for the VHS airborne SAR imaging, we propose an extended MAM (EMAM) method, as shown in Figure 2b. Compared with the IMAM method, the EMAM method realizes higher accuracy by further estimating and compensating the second-order component of the spatially-dependent Doppler rate and the first-order component of the spatially-dependent derivative of the Doppler rate. The former is to avoid the azimuth sidelobe lifting, and the latter is to get rid of the azimuth sidelobe asymmetry. Specifically, the new EMAM method firstly achieves sub-view images via multi-looking processing. Then, a sliding-window-based cross-correlation is implemented to achieve image offsets. Based on the unique mapping between such offset and the Doppler parameters, the DPEC can be accurately implemented.

The paper is arranged as follows. Section 2 introduces the basic MAM method. Section 3 derives the new EMAM method. Section 4 discusses the performance of the proposed method. In Section 5, the validity of the proposed method is verified based on the computer simulations and the real airborne data. Section 6 summarizes this study.

## 2. Basic Multiple Aperture Mapdrift Method

The core strategy of the MAM method divides the data along the azimuth into multiple blocks and generates multiple sub-view images. By searching the offsets between two different sub-view images, it is possible to estimate the higher order Doppler parameters. The MAM methods can be implemented via either azimuth frequency-domain blocking [1] or time-domain blocking [22]. Specifically, the data are divided into several parts in azimuth in the time domain after multiplying the deramping function. Then, the azimuth fast Fourier transform (FFT) is carried out individually for each part to achieve multiple sub-view images. As the presented EMAM method is an extension of the basic MAM method, it is necessary to firstly give a brief introduction to the basic MAM method as follows.

Assume that the signal at a certain range cell is expressed as (1) (ignoring the azimuth four-order and higher order terms of the phase).

(1) $$s\left(t_{a}\right)=\mathrm{rect}\left(\frac{t_{a}}{T_{s}}\right)\exp\left(j2\pi f_{dc}t_{a}+j\pi f_{dr,a}t_{a}^{2}+j\pi f_{3rd,a}t_{a}^{3}\right),$$

where $f_{dc}$ is the Doppler centroid. $T_{s}$ is the azimuth accumulation time. $f_{dr,a}$ and $f_{3rd,a}$ represent the real Doppler rate and the derivative of the Doppler rate, respectively. $t_{a}$ is the azimuth slow time.

The deramping function is described as (2).

(2) $$s\left(t_{a}\right)=\mathrm{rect}\left(\frac{t_{a}}{T_{s}}\right)\exp\left(-j\pi f_{dr,b}t_{a}^{2}-j\pi f_{3rd,b}t_{a}^{3}\right),$$

where $f_{dr,b}$ and $f_{3rd,b}$ represent the calculated Doppler rate and the derivative of the Doppler rate, respectively, which are inaccurate.

After being multiplied by the deramping function, the data are as follows.

(3) $$s\left(t_{a}\right)=\mathrm{rect}\left(\frac{t_{a}}{T_{s}}\right)\exp\left(j2\pi f_{dc}t_{a}+j\pi e_{dr}t_{a}^{2}+j\pi e_{3rd}t_{a}^{3}\right),$$

where $e_{dr}$ and $e_{3rd}$ represent the errors of the Doppler rate and the derivative of the Doppler rate, respectively.

Then, the data are divided into three equal long sub-segments as (4).

(4) $$s_{i}\left(t_{a}\right)=\mathrm{rect}\left(\frac{t_{a}-t_{ac,i}}{T_{s}/3}\right)\exp\left(j2\pi f_{dc}t_{a}+j\pi e_{dr}t_{a}^{2}+j\pi e_{3rd}t_{a}^{3}\right),$$

where $t_{ac,i}$ is the azimuth time for the center of each sub-segment and can be expressed as follows.

(5) $$t_{ac,i}=-\frac{T_{s}}{3}+\left(i-1\right)\frac{T_{s}}{3},\;i=1,2,3.$$

The data in (4) can be translated to the position where $t_{a}=0$ and $t_{a}$ is replaced by $t_{a}+t_{ac,i}$.

(6) $$s_{i}\left(t_{a}\right)=\mathrm{rect}\left(\frac{t_{a}}{T_{s}/3}\right)\exp\left\{\begin{array}{c} j2\pi f_{dc}\left(t_{a}+t_{ac,i}\right)\\ +j\pi e_{dr}{\left(t_{a}+t_{ac,i}\right)}^{2}\\ +j\pi e_{3rd}{\left(t_{a}+t_{ac,i}\right)}^{3} \end{array}\right\}.$$

By performing the phase derivative of the upper formula and letting $t_{a}=0$, the coefficient of the first-order phase can be obtained as (7).

(7) $$f_{d,i}=f_{dc}+e_{dr}t_{ac,i}+\frac{3}{2}e_{3rd}t_{ac,i}^{2}.$$

Three sub-segments are subjected to the azimuth FFT to obtain three sub-view images, respectively. The center of sub-i image is located at $f_{d,i}$, and the position offset between sub-i image and sub-j image can be expressed as (8).

(8) $$\Delta f_{d,ij}=f_{d,i}-f_{d,j}=e_{dr}\left(t_{ac,i}-t_{ac,j}\right)+\frac{3}{2}e_{3rd}\left(t_{ac,i}^{2}-t_{ac,j}^{2}\right).$$

Then, three pairs of sub-view images can be formed to get three position offsets. The system of equations is as follows.

(9) $$\Delta f=T_{ac}\left[\begin{array}{c} e_{dr}\\ \frac{3}{2}e_{3rd} \end{array}\right],$$

where:

$$\Delta f={\left[\Delta f_{d,12}\;\Delta f_{d,13}\;\Delta f_{d,23}\right]}^{T}$$

(10) $$T_{ac}=\left[\begin{array}{ccc} t_{ac,1}-t_{ac,2} & t_{ac,1}^{2}-t_{ac,2}^{2} & t_{ac,1}^{3}-t_{ac,2}^{3}\\ t_{ac,1}-t_{ac,3} & t_{ac,1}^{2}-t_{ac,3}^{2} & t_{ac,1}^{3}-t_{ac,3}^{3}\\ t_{ac,2}-t_{ac,3} & t_{ac,2}^{2}-t_{ac,3}^{2} & t_{ac,2}^{3}-t_{ac,3}^{3} \end{array}\right].$$

After the cross-correlation of two sub-view images is computed and the position of the correlation peak is searched, the estimated value of the offset $\Delta {\hat{f}}_{d,ij}$ between two sub-view images can be obtained, then $\Delta {\hat{f}}_{d,ij}$ is taken into (9) to estimate the errors of the Doppler parameters by the least squares principle.

(11) $$\left[\begin{array}{c} {\hat{e}}_{dr}\\ \frac{3}{2}{\hat{e}}_{3rd} \end{array}\right]={\left(T_{ac}^{T}T_{ac}\right)}^{-1}T_{ac}^{T}\Delta \hat{f}.$$

In practice, since the sub-view images are defocused and the defocus conditions of the different sub-view images are not exactly the same, there is certain error in the position of correlation peak of the sub-view image, so the MAM methods often require multiple iterations to achieve better estimate accuracy.

It can be seen that the basic MAM method only compensates the spatially-invariant Doppler phases and hence can be only applied for the broadside or small-squint cases. Although the IMAM methods have partly overcome this disadvantage by estimating and compensating the spatial reliance of the Doppler phase up to the second-order, they still suffer from the problem of insufficient accuracy for the VHS SAR imaging. In this study, this problem is solved by further estimating and compensating the spatial variance of the third-order Doppler phase, resulting in the new EMAM method.

## 3. Extended Multiple Aperture Mapdrift Method

The spatial variance of the Doppler parameters refers to the fact that these parameters change with the azimuth position of target and can be represented as the functions of $f_{dc}$. Thus, the errors of the Doppler rate $e_{dr}$ and the derivative of the Doppler rate $e_{3rd}$ in (3) become the functions of $f_{dc}$, i.e., $e_{dr}\left(f_{dc}\right)$ and $e_{3rd}\left(f_{dc}\right)$. The offset of two sub-view images in (8) also becomes the function of $f_{dc}$ as shown in (12).

(12) $$\Delta f_{d,ij}\left(f_{dc}\right)=e_{dr}\left(f_{dc}\right)\left(t_{ac,i}-t_{ac,j}\right)+\frac{3}{2}e_{3rd}\left(f_{dc}\right)\left(t_{ac,i}^{2}-t_{ac,j}^{2}\right).$$

**Figure 3:**
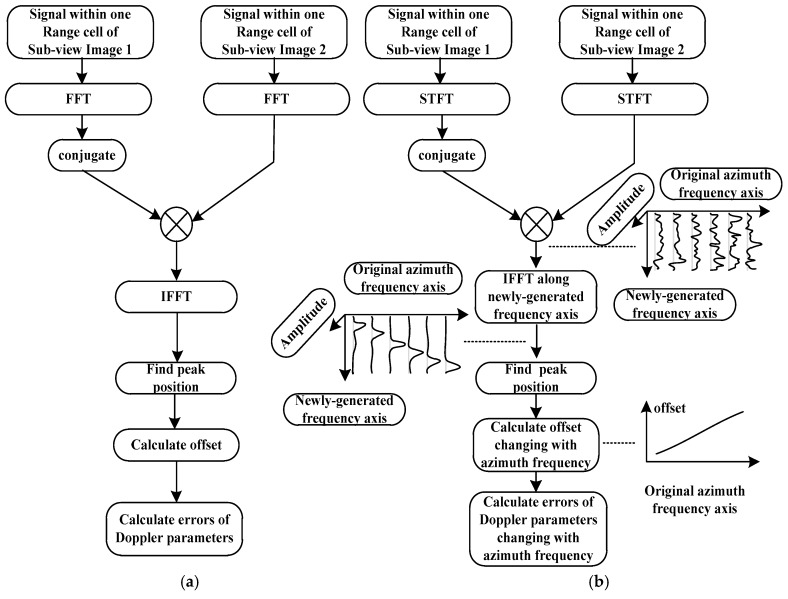
The flow charts of the basic MAM method and the EMAM method. (**a**) The basic MAM method; (**b**) the EMAM method.

Then, an additional sliding windowing manipulation for the sub-view image correlation is employed to obtain the corresponding image offset $\Delta f_{d,ij}\left(f_{dc}\right)$. Specifically, the sliding windowing manipulation is implemented by the short time Fourier transform (STFT). The two sub-view images at the same range cell are individually processed by the STFT, followed by the conversion of the data dimension from one to two, where one denotes the original Doppler frequency and the other denotes the newly-generated frequency. After the conjugate multiplication of the data, the IFFT is generated along the new frequency axis. Then, the offset of the sub-view images can be obtained based on the peak position. Figure 3 shows the flowcharts of the basic MAM method and the EMAM method. It can be seen that the use of STFT can achieve the sliding windowing manipulation, and the Doppler parameters changing with the azimuth frequency can be obtained. In order to improve the efficiency in practical applications, the intervals between windows can be appropriately increased, and the offset of each azimuth frequency can be obtained by the curve fitting. Then, the spatially-variant ${\hat{e}}_{dr}\left(f_{dc}\right)$ and ${\hat{e}}_{3rd}\left(f_{dc}\right)$ can be obtained based on the estimated $\Delta {\hat{f}}_{d,ij}\left(f_{dc}\right)$.

After obtaining ${\hat{e}}_{dr}\left(f_{dc}\right)$ and ${\hat{e}}_{3rd}\left(f_{dc}\right)$, the operation of the curve fitting is performed. Here, the quadratic curve fitting is taken as an example.

(13) $${\hat{e}}_{dr}\left(f_{dc}\right)=e_{dr0}+e_{dr1}\left(f_{dc}-f_{dc,cen}\right)+e_{dr2}{\left(f_{dc}-f_{dc,cen}\right)}^{2}\;{\hat{e}}_{3rd}\left(f_{dc}\right)=e_{3rd0}+e_{3rd1}\left(f_{dc}-f_{dc,cen}\right)+e_{3rd2}{\left(f_{dc}-f_{dc,cen}\right)}^{2},$$

where $f_{dc,cen}$ is the Doppler centroid of the azimuth center of the scene, the first terms of the two expressions are the fixed errors, the second terms are the first-order spatial variance errors, and the third terms are the second-order spatial variance errors. In general, the second-order spatial variance error of the derivative of the Doppler rate is too small to be ignored. The first- and second-order spatial variance errors of the Doppler rate and the first-order spatial variance error of the derivative of the Doppler rate should be estimated and compensated.

**Figure 4:**
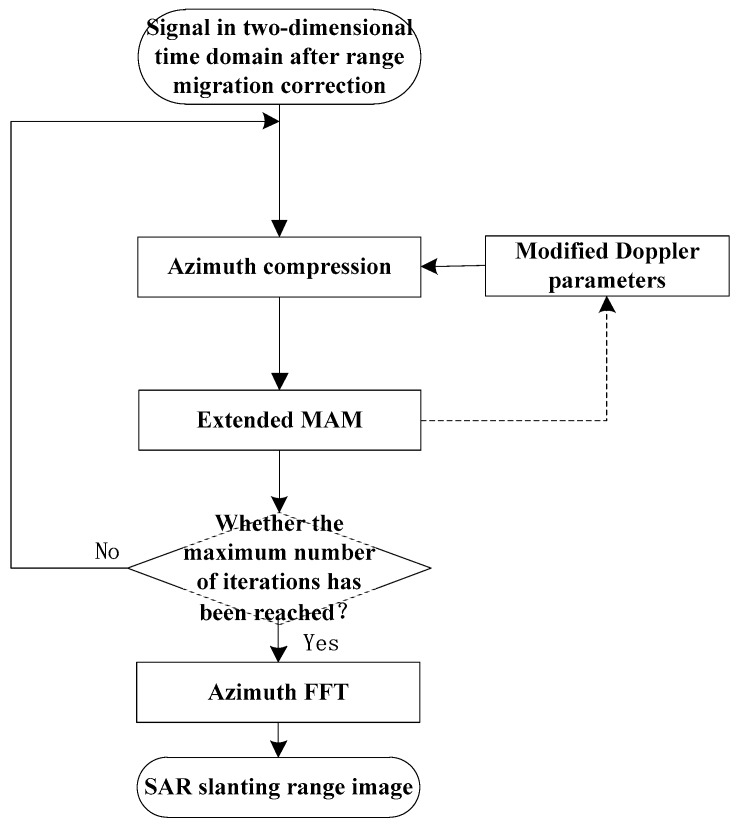
The flowchart of the azimuth compression combined with the EMAM method in the high-squint airborne SAR imaging algorithm.

Use the fitting coefficients in (13) to correct the corresponding Doppler parameters in the high-squint airborne SAR imaging algorithm [6] so as to achieve the focus improved image. In order to improve the accuracy of the Doppler parameter estimation, multiple iterations are performed. The flowchart of the azimuth compression combined with the EMAM method in the high-squint SAR imaging algorithm is shown in Figure 4.

## 4. Performance Analysis

### 4.1. Spatial Variance of Doppler Parameters

**Figure 5:**
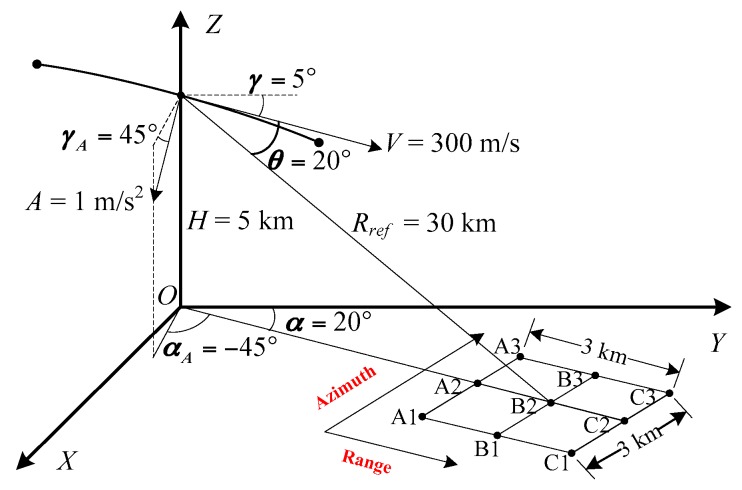
Typical VHS airborne SAR geometry.

The spatial variance of the Doppler parameters is analyzed based on a typical VHS airborne SAR geometry, as shown in Figure 5. The XOY plane is the ground plane. V and A are the velocity and acceleration of the aircraft. The velocity vector is in the YOZ plane. H is the aircraft altitude. $R_{ref}$ is the corresponding slanting distance. γ and $\gamma_{A}$ are the velocity dive angle (between the velocity vector and the horizontal plane) and the acceleration dive angle (between the acceleration vector and the horizontal plane), respectively. α and $\alpha_{A}$ are the velocity azimuth angle (between the projections of the slanting distance vector and the velocity vector to the ground) and the acceleration azimuth angle (between the projections of the slanting distance vector and the acceleration vector to the ground), respectively. θ is the squint angle (between the velocity vector and the slanting distance vector). The aircraft motion time is 4 s.

**Figure 6:**
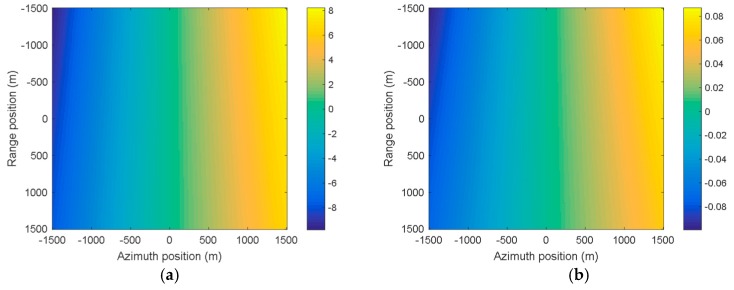
The spatial variance of the Doppler parameters. (**a**) The Doppler rate; (**b**) the derivative of the Doppler rate.

**Figure 7:**
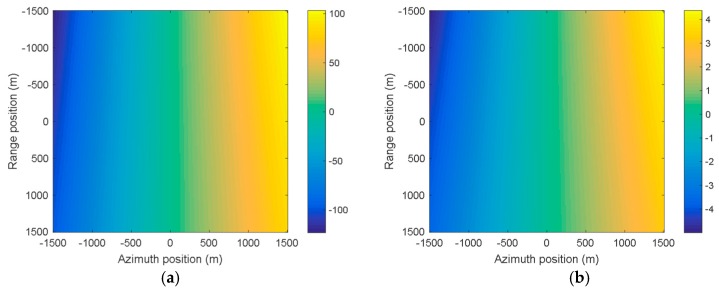
The spatially-variant phase errors caused by the spatially-variant Doppler parameters. (**a**) The phase error caused by the Doppler rate; (**b**) the phase error caused by the derivative of the Doppler rate.

Figure 6a,b shows the spatial variance of the Doppler rate and the derivative of the Doppler rate, respectively. The center of the figure represents the beam irradiation position B2. It can be clearly seen that the spatial variations of the Doppler rate and the derivative of the Doppler rate are about 8 Hz/s and $0.08{\;\mathrm{Hz}}^{3}$, respectively. Figure 7a,b shows the spatially-variant phase errors caused by the spatially-variant Doppler parameters, which are about 100 rad (larger than $\frac{\pi }{4}$) and 4 rad (larger than $\frac{\pi }{8}$), respectively. If the phase error caused by the Doppler rate is larger than $\frac{\pi }{4}$, or the phase error caused by the derivative of the Doppler rate is larger than $\frac{\pi }{8}$, it will seriously degrade the image quality. Thus, the spatial variance of the Doppler rate and the derivative of the Doppler rate should be estimated and compensated.

The data at the range time domain and the azimuth frequency domain in the step of the azimuth compression for the high-squint airborne SAR imaging algorithm is as follows.

(14) $$s\left(f_{a},t_{r};R,\theta \right)=\sin c\left(\frac{t_{r}-2R/c}{1/B_{r}}\right)\mathrm{rect}\left(\frac{f_{a}-f_{ac}}{f_{dr}T_{s}}\right)\exp\left\{j\varphi_{0}+j\pi a_{2}{\left(f_{a}-f_{ac}\right)}^{2}+j\pi a_{3}{\left(f_{a}-f_{ac}\right)}^{3}\right\},$$

where the first sinc function is the result of the range pulse compression, and the latter two are the azimuth envelope and phase modulations. $t_{r}$ and $f_{a}$ are the range time and the azimuth frequency, respectively. $f_{ac}$ and $f_{dr}$ are the Doppler centroid and the Doppler rate of the target with the slant range R and the squint angle θ (which is the angle between the velocity vector and range vector), respectively. $B_{r}$ is the signal bandwidth. $T_{s}$ is the azimuth accumulation time. In the phase modulation, the constant phase $\varphi_{0}$ does not affect focus, and the spatial variance of $a_{2}$ and $a_{3}$ (related to the spatial variance of the Doppler parameters) is analyzed below. $a_{2}$ and $a_{3}$ can be expressed as the functions of $\left(R,f_{dc}\right)$. Then, these functions can be further expanded at $f_{dc}=f_{dc,cen}=\left(2V\cos\theta_{cen}\right)/\lambda$ as the Taylor series shown in (15) [6].

(15) $$a_{2}\approx a_{20}+a_{21}\left(f_{dc}-f_{dc,cen}\right)+a_{22}{\left(f_{dc}-f_{dc,cen}\right)}^{2}\;a_{3}\approx a_{30}+a_{31}\left(f_{dc}-f_{dc,cen}\right),$$

where $a_{20}$ and $a_{30}$ are the constant coefficients, $a_{21}$ and $a_{31}$ are the first-order spatial variance coefficients, and $a_{22}$ is the second-order spatial variance coefficient of $a_{2}$.

When the target is at the azimuth center of the distance-isoline of the illuminated scene, θ and $f_{ac}$ become $\theta_{cen}$ and $f_{dc,cen}$, respectively. V is the aircraft velocity. λ is the wavelength.

In order to get a well-focused image, the absolute values of the phase errors caused by the spatial variance of $a_{2}$ and $a_{3}$ should be less than $\frac{\pi }{4}$ and $\frac{\pi }{8}$, as expressed by (16) and (17), respectively.

(16) $$\left|a_{21}\left(f_{dc}-f_{dc,cen}\right){\left(\frac{B_{a}}{2}\right)}^{2}\pi \right|<\frac{\pi }{4},\;\left|a_{22}{\left(f_{dc}-f_{dc,cen}\right)}^{2}{\left(\frac{B_{a}}{2}\right)}^{2}\pi \right|<\frac{\pi }{4},$$

(17) $$\left|a_{31}\left(f_{dc}-f_{dc,cen}\right){\left(\frac{B_{a}}{2}\right)}^{3}\pi \right|<\frac{\pi }{8},$$

where $B_{a}$ is the Doppler width of the target.

### 4.2. Complexity

The computational complexity (floating-point operation) of the EMAM method is analyzed in detail. For the signal at a certain range cell, the number of azimuth points is $N_{a}$. The complexity of the main steps of the EMAM method is as shown in Table 1.

Therefore, the computational complexity of the main steps for the EMAM method can be written as (18).

(18) $$S_{\mathrm{com}}=S_{I}+S_{\mathrm{II}}+S_{\mathrm{III}}.$$

In order to improve the accuracy of the Doppler parameters, multiple iterations are performed. Therefore, if the number of iterations is K, the computational complexity of the EMAM method can be written as:

(19) $$S_{\mathrm{EMAM}}=KS_{\mathrm{com}}.$$

The complexity of the main steps of the basic MAM method and the IMAM method are shown in Table 2 and Table 3, respectively.

**Figure 8:**
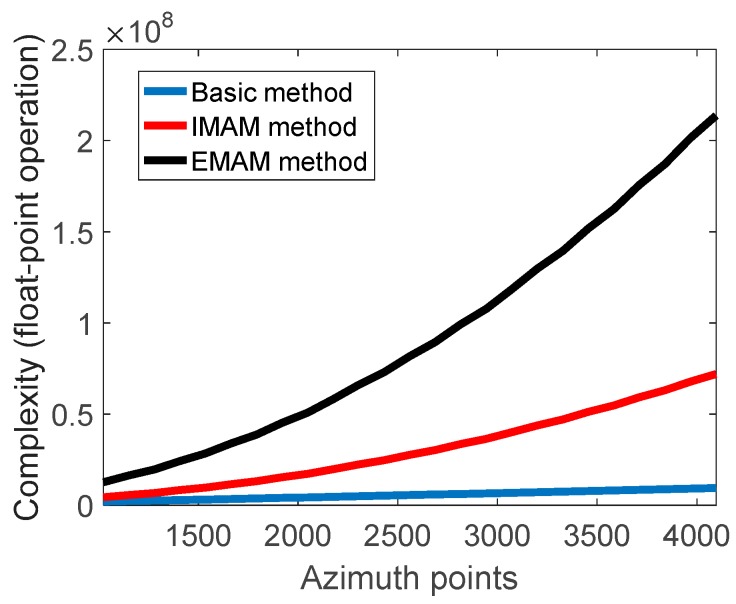
The computational complexity of the different methods.

Assuming that the number of iterations K is three, the window width $N_{w}$ in the IMAM method and the EMAM method is 100, and the number of sliding windowing manipulations L is $N_{a}/50$, then the complexity of the different methods can be compared as shown in Figure 8. It can be clearly seen that the complexity of the EMAM method is larger than the basic method and the IMAM method due to further estimating and compensating the first-order component of the spatially-dependent derivative of the Doppler rate, which increases the data processing time. When $N_{a}$ is 4096, the complexities of the basic method, the IMAM method, and the EMAM method are $9.437\times 10^{6}$, $7.196\times 10^{7}$, and $2.136\times 10^{8}$, respectively. However, the computational complexity of the proposed method does not change qualitatively, and the real-time implementation of the EMAM onboard could be achieved after evaluating the existing hardware systems.

## 5. Results

### 5.1. Simulation

The point target simulations are performed based on the geometry in Figure 5. The point targets are distributed as a 3×3 matrix on the ground plane with both 3 km in range and azimuth. In order to illustrate the advantages of the EMAM method, the imaging results of the basic MAM method and the IMAM method are given. The velocity error ΔV(10 m/s) and the acceleration error $\Delta A\left(-0.1\;m/s^{2}\right)$ are added in the imaging process. Here are the examples of point targets C1, C2, and C3 in Figure 5 to illustrate and compare the estimation results of the Doppler parameters of the different methods.

**Figure 9:**
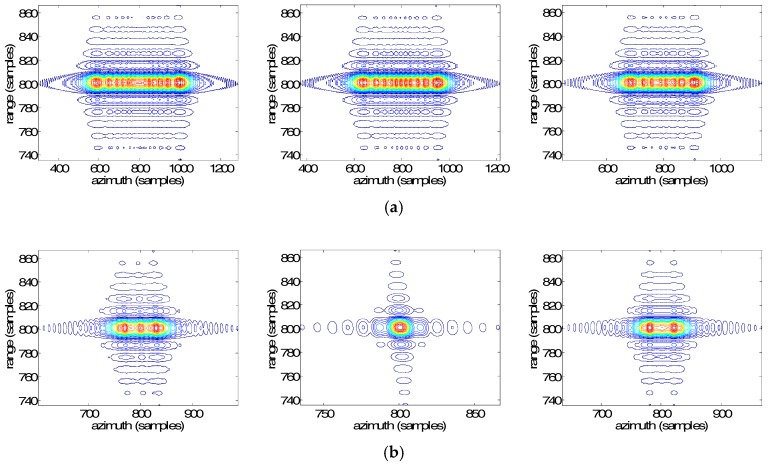

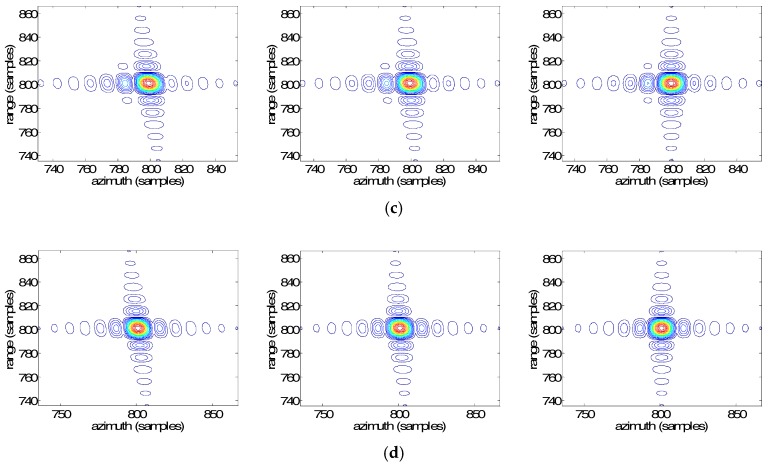
The two-dimensional imaging results of targets by the different methods with the velocity and acceleration errors. (**a**) No Doppler parameter estimation; (**b**) the basic MAM method; (**c**) the IMAM method; (**d**) the EMAM method.

**Figure 10:**
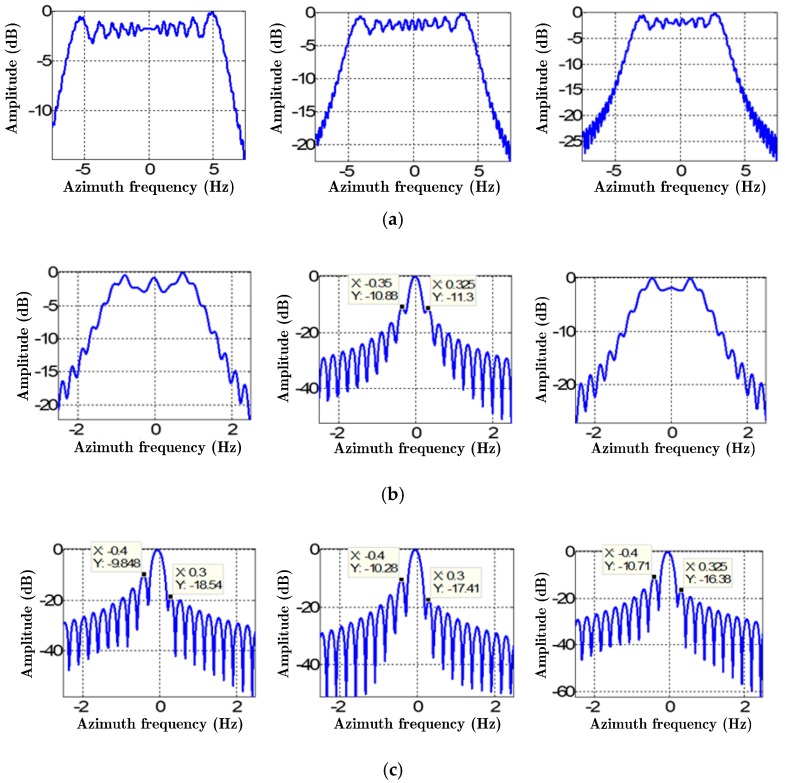

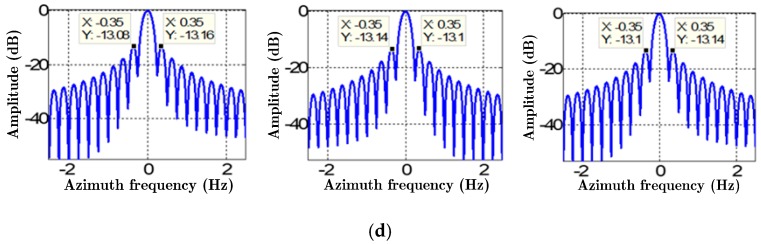
The azimuth impulse responses of targets by the different methods with the velocity and acceleration errors. (**a**) No Doppler parameter estimation; (**b**) the basic MAM method; (**c**) the IMAM method; (**d**) the EMAM method.

Figure 9 and Figure 10 show the two-dimensional imaging results and the azimuth impulse responses of targets by the different methods with the velocity and the acceleration errors, respectively. In Figure 9, the horizontal axis and the vertical axis represent the azimuth samples and range samples, respectively. In Figure 10, the horizontal axis represents the azimuth frequency and the vertical axis refers to the corresponding amplitude of the target (converted to dB). The sub-images from left to right represent C1, C2, and C3 in turn. Figure 9a and Figure 10a show the two-dimensional imaging results and the azimuth impulse responses based on the Doppler parameters with errors, respectively, and there is no Doppler parameter estimation. It can be clearly seen that the images are seriously defocused, and the Doppler bandwidths of the three points after the deramping are still about 10 Hz, indicating that there are still significant secondary phases, and the errors of the Doppler rates are very large. Figure 9b and Figure 10b show the two-dimensional imaging results and the azimuth impulse responses by the basic MAM method, respectively. It can be seen that the focus of point target C2 at the azimuth center is better, but point targets C1 and C3 at the azimuth edges are noticeably defocused. The main reason is that the estimated Doppler parameters by the basic MAM method are the averaged results of the real ones, and their spatial variance is not considered, resulting in the fact that the point targets at the azimuth edges still have significant secondary phase errors. Figure 9c and Figure 10c show the results of the IMAM method. It can be seen that the sidelobes of the three point targets are asymmetrical because the IMAM method only deals with the spatial variance of the Doppler rate. The peak sidelobe ratios are about −10 dB, as shown in Table 4. Figure 9d and Figure 10d show the results of the EMAM method. It can be seen that the targets both at the azimuth center and edges are well-focused, indicating that the EMAM method can estimate the spatial variance of the Doppler rate and the derivative of the Doppler rate well. The peak sidelobe ratios are about −13 dB, as shown in Table 4, indicating that the EMAM method has achieved higher estimation accuracy.

In order to further illustrate the accuracy of the Doppler parameter estimation, Table 5 shows the estimation results of the errors of the Doppler parameters based on the basic MAM method, the IMAM method, and the EMAM method. It can be seen that the estimation results of ${\hat{e}}_{dr0}$ based on the three methods are relatively close to the real values, and likewise for the estimation results of ${\hat{e}}_{3rd0}$ by the basic method and the EMAM method. The IMAM method and the EMAM method can estimate ${\hat{e}}_{dr1}$ well. However, the errors of the estimation results of ${\hat{e}}_{dr2}$ based on the IMAM method and the EMAM method are relatively large. The reason is that the phase error caused by this term is very small and has little effect on the image focus based on the specific geometry in Figure 5. Moreover, the EMAM method can further estimate ${\hat{e}}_{3rd1}$ well.

### 5.2. Real Data

**Figure 11:**
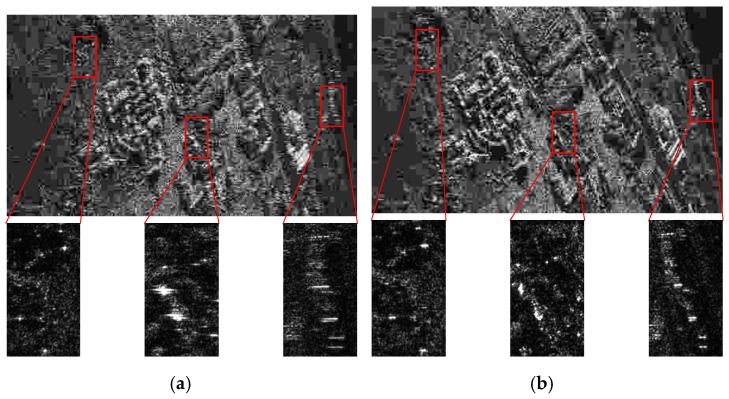

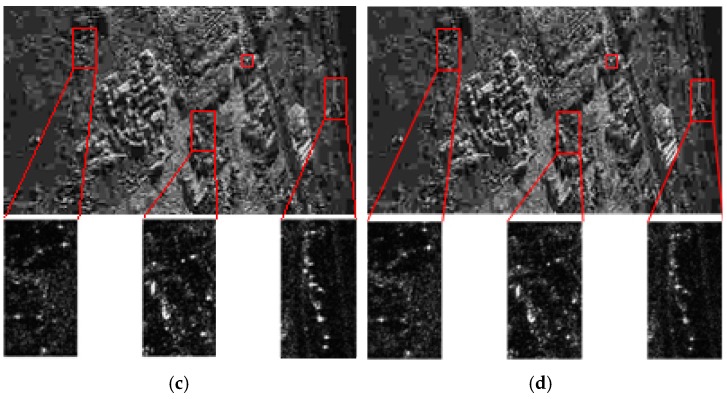
The images of real airborne data based on the different methods. (**a**) The inertial navigation information; (**b**) the basic MAM method; (**c**) the IMAM method; (**d**) the EMAM method.

To validate the EMAM method in practical applications, this section gives the results of real airborne SAR data based on the different methods. The velocity of the aircraft is about 105 m/s, and the acceleration is about $0.26\;m/s^{2}$. The aircraft altitude is about 5 km. The squint angle is about 30°. The azimuth width of the image is about 1.2 km, and the range width is about 500 m. The data are processed based on the inertial navigation information (inaccurate), the basic MAM method, the IMAM method, and the EMAM method, respectively, and the results of the slanting distance image are shown in Figure 11. In the figures, the horizontal direction represents the azimuth frequency domain, and the vertical direction refers to the range time domain.

**Figure 12:**
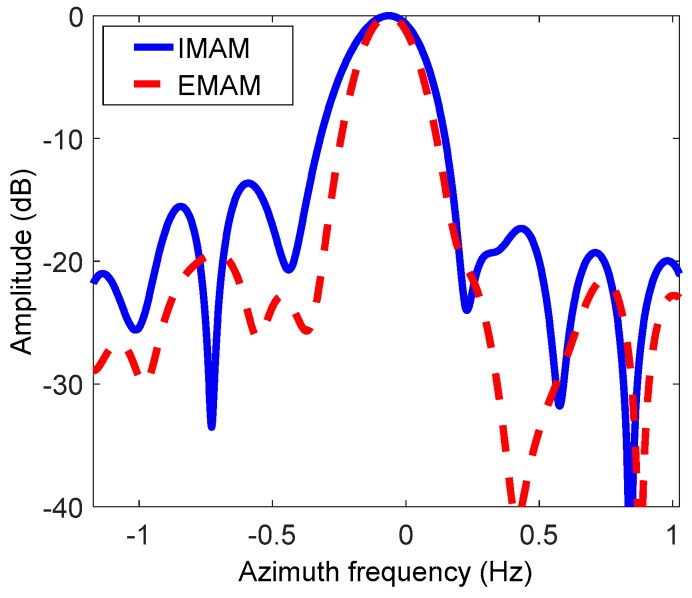
The azimuth impulse responses of the chosen strong scatterer based on the IMAM method and the EMAM method.

Figure 11a is the image based on the inertial navigation information. It can be seen that the defocus condition of the image is more and more serious from left to right, indicating that the spatial variance of the Doppler parameters is very obvious. The Doppler parameters calculated from the inertial information are closer to the real ones of the left scene. Figure 11b is the image based on the basic MAM method. The azimuth center of the scene is well-focused, but there is still obvious defocus at the azimuth edges, indicating that the estimation results of the Doppler parameters by the basic MAM method are the averages of the real Doppler parameters of the whole scene, which are close to the real ones of the central scene. Figure 11c,d shows the images based on the IMAM method and the EMAM method, respectively. It can be seen that the focus of the image has been significantly improved compared with the basic MAM method, but the comparison between these two methods is not obvious. Therefore, a strong scatterer in the small red square is chosen as shown in Figure 11c,d to further compare the two methods. Figure 12 is the azimuth impulse responses of the chosen strong scatterer based on the IMAM method and the EMAM method. It can be clearly seen that the azimuth sidelobe asymmetry exists in the IMAM result, while for the EMAM result, the main-lobe is narrower and the side-lobe is lower and basically symmetrical, which explains that the EMAM method is better than the IMAM method.

**Figure 13:**
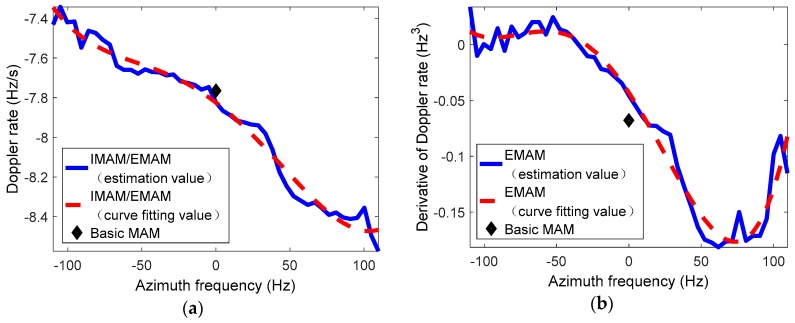
The estimation curves of the spatially-variant Doppler parameters based on the different methods. (**a**) The Doppler rate; (**b**) the derivative of the Doppler rate.

Figure 13 shows the estimation curves of the spatially-variant Doppler parameters based on the different methods. It can be seen from the figures that the Doppler parameters obviously change with the azimuth frequency. The estimation results of the Doppler rate and the derivative of the Doppler rate by the basic MAM method are basically the averages of the EMAM method. The blue solid lines represent the estimation results of the Doppler parameters by the IMAM method or the EMAM method, and the red dotted lines refer to the curve fitting values of the estimated Doppler parameters. As shown in Figure 13a, the IMAM method can estimate the spatially-variant Doppler rate, and the estimation result is basically consistent with the EMAM method; while the EMAM method can further estimate the spatially-variant derivative of the Doppler rate as shown in Figure 13b.

**Figure 14:**
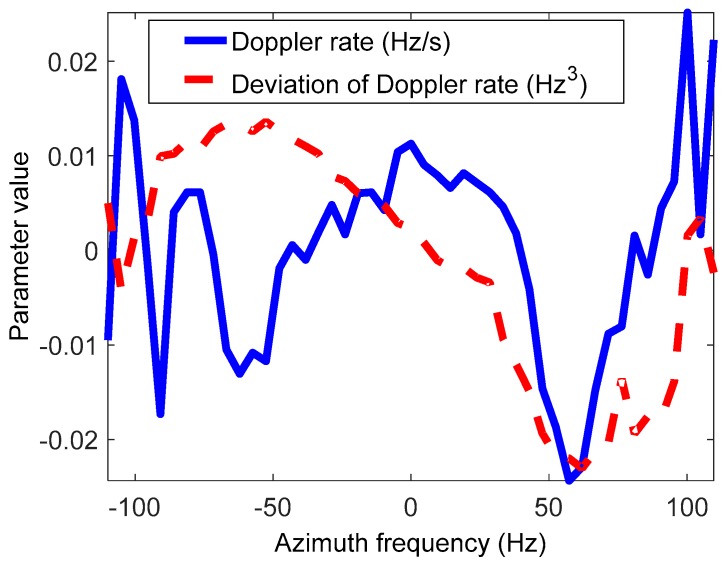
The residual spatial variance of the Doppler parameters after compensation based on the estimation results of the EMAM method.

Figure 14 shows the residual spatial variance of the Doppler parameters after compensation based on the estimation results of the EMAM method. It can be seen that the first- and second-order spatial variance of the Doppler rate and the first-order spatial variance of the derivative of the Doppler rate are basically eliminated; only the higher order spatial variance is left, which does not affect the focus of the image.

## 6. Conclusions

In this study, an EMAM method has been proposed for DPEC of the VHS airborne SAR imaging. Comparing with the existing MAM-based DPEC methods, the EMAM is superior in achieving higher accuracy benefiting from the additional estimation and compensation for the spatial dependence of the third-order Doppler phase, corresponding to the derivative of the Doppler rate. The EMAM method not only avoids the azimuth sidelobe lifting, but also gets rid of the azimuth sidelobe asymmetry. Specifically, the EMAM method firstly achieves sub-view images via multi-looking processing. Then, a sliding-window-based cross-correlation is implemented to achieve image offsets. Based on the unique mapping between such offsets and the Doppler parameters, the DPEC can be accurately implemented. By showing that the EMAM outperforms the existing DPEC methods in both the computer simulations and the real airborne data processing experiments, the effectiveness of the presented approach has been validated. Both the computer simulations and the real airborne data processing experiments show that based on the EMAM method, the targets both at the azimuth center and edges are well focused, indicating that the EMAM method can accurately estimate and compensate the spatial variance of the Doppler rate and the derivative of the Doppler rate. Further research may focus on the real-time implementation of the EMAM onboard.

## Figures and Tables

**Table 1:** The complexity of the main steps of the EMAM method.

Main Step	Operation	Complexity
$S_{I}$: Achieving three sub-view images	FFT	$15N_{a}\log_{2}N_{a}$
$S_{\mathrm{II}}$: Estimating ${\hat{e}}_{dr}\left(f_{dc}\right)$ and ${\hat{e}}_{3rd}\left(f_{dc}\right)$ (L operations of sliding windowing manipulation, window width: $N_{w}$)	STFT	$30LN_{w}\log_{2}N_{w}$
	Complex conjugate multiplication	$21LN_{w}$
	IFFT	$15LN_{a}\log_{2}N_{a}$
	Modulus	$27LN_{a}$
$S_{\mathrm{III}}$: Estimating the fitting coefficients of ${\hat{e}}_{dr}\left(f_{dc}\right)$ and ${\hat{e}}_{3rd}\left(f_{dc}\right)$	Curve fitting	32L
$S_{\mathrm{com}}$: Total	$15N_{a}\log_{2}N_{a}+\left(30N_{w}\log_{2}N_{w}+21N_{w}+15N_{a}\log_{2}N_{a}+27N_{a}+32\right)L$	

**Table 2:** The complexity of the main steps of the basic MAM method.

Main Step	Operation	Complexity
$S_{I}$: Achieving three sub-view images	FFT	$15N_{a}\log_{2}N_{a}$
$S_{\mathrm{II}}$: Estimating ${\hat{e}}_{dr}$ and ${\hat{e}}_{3rd}$	FFT	$30N_{a}\log_{2}N_{a}$
	Complex conjugate multiplication	$21N_{a}$
	IFFT	$15N_{a}\log_{2}N_{a}$
	Modulus	$27N_{a}$
$S_{\mathrm{com}}$: Total	$15N_{a}\log_{2}N_{a}+30N_{a}\log_{2}N_{a}+21N_{a}+15N_{a}\log_{2}N_{a}+27N_{a}$	

**Table 3:** The complexity of the main steps of the improved MAM (IMAM) method.

Main Step	Operation	Complexity
$S_{I}$: Achieving two sub-view images	FFT	$10N_{a}\log_{2}N_{a}$
$S_{\mathrm{II}}$: Estimating ${\hat{e}}_{dr}\left(f_{dc}\right)$ (L operations of sliding windowing manipulation, window width: $N_{w}$)	STFT	$10LN_{w}\log_{2}N_{w}$
	Complex conjugate multiplication	$7LN_{w}$
	IFFT	$5LN_{a}\log_{2}N_{a}$
	Modulus	$9LN_{a}$
$S_{\mathrm{III}}$: Estimating the fitting coefficients of ${\hat{e}}_{dr}\left(f_{dc}\right)$	Curve fitting	16L
$S_{\mathrm{com}}$: Total	$10N_{a}\log_{2}N_{a}+\left(10N_{w}\log_{2}N_{w}+7N_{w}+5N_{a}\log_{2}N_{a}+9N_{a}+16\right)L$	

**Table 4:** The azimuth performance analysis of the three point targets C1, C2, and C3 based on the basic MAM, the IMAM method, and the EMAM method.

Method	Index	Point Target C1	Point Target C2	Point Target C3
**Basic MAM**	PSLR (dB)	−4.72	−10.88	−6.12
	ISLR (dB)	−8.25	−7.87	−8.72
	Azimuth resolution (m)	5.67	0.59	4.35
**IMAM**	PSLR (dB)	−9.85	−10.28	−10.71
	ISLR (dB)	−8.69	−8.95	−9.18
	Azimuth resolution (m)	0.59	0.58	0.58
**EMAM**	PSLR (dB)	−13.08	−13.10	−13.10
	ISLR (dB)	−9.63	−9.64	−9.63
	Azimuth resolution (m)	0.57	0.57	0.57

Note: PSLR represents the peak sidelobe ratio (the peak strength ratio of the highest side-lobe to the main-lobe), and ISLR represents the integral sidelobe ratio (the energy radio of all side-lobes to the main-lobe). The theoretical azimuth resolution of the three point targets is 0.57 m.

**Table 5:** The estimation results of the errors of the Doppler parameters by the different methods.

Error Coefficient	Real Value	Basic MAM	IMAM	EMAM
${\hat{e}}_{dr0}\;\left(\mathrm{Hz}/s\right)$	−2.6426	−2.7048	−2.6485	−2.6464
${\hat{e}}_{dr1}\;\left(\mathrm{Hz}\right)$	0.0012	-	0.0012	0.0012
${\hat{e}}_{dr2}$	1.2575 × 10^−7^	-	1.4614 × 10^−7^	1.5608 × 10^−7^
${\hat{e}}_{3rd0}\;\left({\mathrm{Hz}}^{3}\right)$	−0.0360	−0.0396	-	−0.0390
${\hat{e}}_{3rd1}\;\left({\mathrm{Hz}}^{2}\right)$	1.2540 × 10^−5^	-	-	1.2630 × 10^−5^

Note: “-” indicates that the basic MAM method or the IMAM method cannot estimate this error coefficient. ${\hat{e}}_{dr2}$ is non-dimensional.
